# Dichotomous role of autophagy in cancer

**DOI:** 10.2478/abm-2022-0014

**Published:** 2022-06-30

**Authors:** Amin Arif, Muhammad Babar Khawar, Rabia Mehmood, Muddasir Hassan Abbasi, Nadeem Sheikh

**Affiliations:** Institute of Zoology, University of the Punjab, Lahore 54000, Pakistan; Department of Zoology, University of Narowal, Narowal 51750, Pakistan; Department of Zoology, University of Okara, Okara 56130, Pakistan

**Keywords:** autophagy, carcinogenesis, neoplasms, pathology, recycling therapeutics

## Abstract

Autophagy is an evolutionary conserved catabolic process that plays physiological and pathological roles in a cell. Its effect on cellular metabolism, the proteome, and the number and quality of organelles, diversely holds the potential to alter cellular functions. It acts paradoxically in cancer as a tumor inhibitor as well as a tumor promoter. In the early stage of tumorigenesis, it prevents tumor initiation by the so-called “quality control mechanism” and suppresses cancer progression. For late-staged tumors that are exposed to stress, it acts as a vibrant process of degradation and recycling that promotes cancer by facilitating metastasis. Despite this dichotomy, the crucial role of autophagy is evident in cancer, and associated with mammalian targets of rapamycin (mTOR), p53, and Ras-derived major cancer networks. Irrespective of the controversy regarding autophagic manipulation, promotion and suppression of autophagy act as potential therapeutic targets in cancer treatment and may provide various anticancer therapies.

Autophagy is a process of so-called self-cannibalism of cytoplasmic contents, including biomolecules and damaged organelles, by lysosomal enzymes to alleviate stress in response to starvation, tumorigenesis, and cell death [1, 2]. Generally, it operates steadily, but various types of intrinsic stress factors, such as nutrient deprivation, involvement of growth factors, DNA damage, abnormal protein accumulation, and worn-out organelles have the potential to induce a higher level or rate of autophagy. These cellular stress factors stimulate the cells to adapt to harsh conditions or to trigger apoptotic processes to eliminate damaged and potentially harmful cells [3]. Low-level autophagy prevents the gradual rise of denatured proteins and cellular organelles that may become harmful. This lysosomal-mediated self-degradation maintains cell survival and metabolism during stress, and eliminates denatured proteins and worn-out organelles. Thus, it is particularly important in preserving the quality of cellular proteins and organelles [4].

Autophagy can be categorized into 3 distinct types: macroautophagy, microautophagy, and chaperone-mediated autophagy (CMA) [5]. The term autophagy usually refers to macroautophagy, which maintains cellular homeostasis via lysosomal degradation of cytoplasmic organelles and other cellular contents. It is regulated by various autophagy-related genes (ATGs) [6] and characterized by the development of phagophores and engulfment of cytoplasmic cargo in a selective or unselective manner. The phagophores, upon maturation, are packed into autophagosomes, which eventually merge with lysosomes to degrade the confined cargo. Ultimately, the degraded material is reprocessed via cellular metabolic reactions [7]. The second type, microautophagy, is the infolding of the lysosomal membrane to engulf cytoplasmic elements, followed by the sealing of the membranous sacs, which are later degraded in the lumen of the lysosome [8]. In microautophagy, entire cell organelles, such as peroxisomes, can be engulfed directly into lysosomes. In CMA, the third type of autophagy, cytoplasmic proteins bearing KFERQ- or KFERQ-like sequence motifs collaborate with heat shock cognate 70 chaperones (HSC70) present in the cytosol and the lysosome-associated membrane glycoprotein type 2A (LAMP2A) to transport the proteins toward lysosomal membranes [9]. Hence, CMA empowers degradation of targeted proteins prohibited by nonspecific or “bulk” protein degradation during macroautophagy.

## Methods

We used PubMed, Google Scholar, and Scopus as primary online databases to search the literature related to this review without limitations as to publication date or language filters, but preference was set to cite the references published in the past 5 years and in the English language. The reference lists of the discovered articles were screened to find articles that were not found by the database searches.

To find the literature relevant to autophagy, the keyword “autophagy” was used. To elaborate its role in cancer, the keywords “tumorigenesis” and “cancer” were used. Keywords “autophagy inhibitor” and “autophagy inducer” were used to highlight the dichotomous role of autophagy in cancer.

We arranged our collected material using the subtitles designated in the review and further cross-verified each sub-heading content with suitable keywords. The search results were imported into EndNote software. Finally, we complied summaries of the literature into a synthesis of our own perspective of how autophagy is collated with cancer therapy. The data were tabulated and explained qualitatively with diagrams created using BioRender.com.

### Mechanism of autophagy

**Figure 1:**
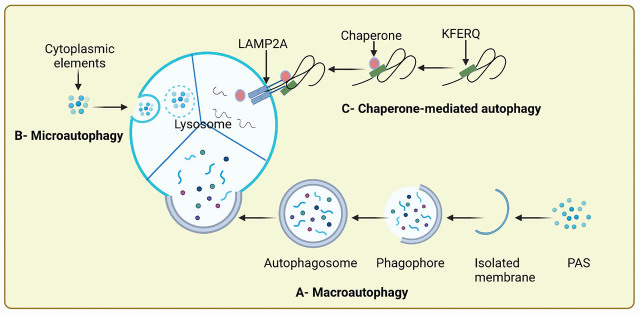
Schematic of autophagic routes. (A) Macroautophagy (commonly named autophagy): the autophagosome comprising several cytosolic polypeptides and proteins merges with lysosomes; subsequently, the autophagosomal content is digested by the lysosomal enzymes. (B) Microautophagy: the cytoplasmic elements directly enclosed by the inward pinching of lysosomal membrane. (C) CMA: the selective components move toward the lysosome after intermingling with the chaperone HSC70 and degrade in the lysosome. Created with BioRender.com. Academic License QJ23ZNIZN0. CMA, chaperone-mediated autophagy; PAS, preautophagosomal structure; HSC70, heat shock cognate 70 chaperones; LAMP2A, lysosome-associated membrane glycoprotein type 2A.

Autophagy is a normal physiological self-degradative process, organized into several steps: induction of autophagy, vesicle nucleation, elongation and maturation of autophagosome, degradation of the autophagosome, and recycling (**Figure 1**) [10].

### Induction of autophagy

The molecular signal of autophagy induction involves 2 regulatory pathways: an energy sensor AMP-activated protein kinase (AMPK) [11] and a cellular nutrient sensor kinase mTOR [12]. Stress such as insufficient glucose level stimulates AMPK. Activated AMPK induces autophagy through different pathways that coincide on the Unc-51 like autophagy activating kinase 1 (ULK1) complex [13]. AMPK also plays an important role in mitophagy and mitochondrial biogenesis [14]. mTOR mediates autophagy in mammals as a major part of regulatory pathway; it affects cellular growth and proliferation in response to growth factors, energy, hypoxia, and nutrient level [15]. mTOR kinase is related to phosphatidylinositol 3-kinase (PI3K) and is an integral component of 2 complexes, mTOR complex 1 (mTORC1) and mTOR complex 2 (mTORC2) [16]. mTORC1 hinders autophagy and regulates cell growth and biogenesis of proteins and lipids. Inhibition of mTORC1 triggers the ULK1 complex that regulates induction of autophagy [17].

Some intrinsic or extrinsic factors stimulate the cells and carry the ULK1 to the preautophagosomal structure (PAS), followed by the hierarchical accretion of autophagy (ATG) proteins. PAS, a dock for the engagement and attachment of ATG proteins, plays a significant role in inducing autophagy. As a result of the ATG docking, the ULK1/ATG1 complex to initiate autophagy is formed, and includes ULK1, ATG13, FIP200, and ATG101. ATG13 of this complex is an important protein for ULK1, which, on the one hand, restricts it to PAS and, on the other, facilitates its interaction with FIP200, while the FIP200 scaffolds ATG protein accretion on the PAS.

### Vesicle nucleation

After inducing autophagy, the ULK1/ATG1 complex starts the process of vesicle nucleation [18]. The PAS, a major nucleating site, forms an isolated membrane, the phagophore, and recruits several ATG proteins. In case of nutrient insufficiency, the ULK1/ATG1 complex couples with ATG13, FIP200, ATG29, and ATG31 to develop a scaffold complex on the PAS. The PI3K complex then settles on the PAS and contributes to phagophore formation through the interaction of ATG14L with the ATG13 at the PAS while ATG9A-positive membrane vesicles that collaborate with the ATG2 WIPI complex are bound to the PAS through their interaction with the FIP200 [19].

### Elongation and maturation of the autophagosome

Multiple ATG proteins interact to form an isolated membrane. First, the small vesicles of ATG9A are merged to synthesize a mortar-shaped phagophore at the PAS, and subsequently the isolated membrane is continuously stretched and wrapped around a chunk of cytoplasmic content or organelles. Finally, the isolated membrane is developed into a mature bilayered membranous structure called the autophagosome. This process is regulated by ATG12-ATG5 and ATG8/LC3 conjugation systems [20].

### Degradation of autophagosome and recycling

The mature autophagosome is then carried to the perinuclear zone for docking and blending with the lysosome [21]. It can mature throughout the cytoplasm indiscriminately; however, lysosomes are restricted to the perinuclear zone. Thus, the autophagosome needs to be transported to the perinuclear zone after maturation, where it blends with lysosomes, unifies into an autophagolysosome, and discriminately degrades proteins and expired organelles into amino acids or peptides with the help of lysosomal hydrolases. This highly coordinated docking and vesicle fusion with targeted components is regulated by the microtubule-associated light chain complex (LC3). Thus, the degraded material becomes available for reuse by cells [22]. To coordinate autophagy, appropriate maturation, and targeted trafficking of the autophagosome as well as lysosomes are prerequisites, and any disruption may stall the process [23].

### Role of autophagy in cancer

Autophagy plays a dynamic and complex role in cancer [24, 25]. It can either promote or suppress tumorigenesis [26, 27]. As a tumor suppressor, autophagy allows cells to eliminate expired cellular contents and consequentially maintain genomic stability, prevent cellular damage, and chronic tissue injury and inflammation. As part of the process, autophagy inhibits the deposition of oncogenic p62 protein in the initial stages of tumorigenesis. Conversely, imperfect autophagy plays a tumor-promoting role because the toxic material contained in cells is not degraded, and consequently, production of reactive oxygen species (ROS), DNA damage, and chromosomal instability follow, reinforcing the survival and resistance of cancerous cells under stress conditions, and ultimately promoting tumorigenesis by supporting tumor metabolism, growth, and survival. So, autophagy is a “double-edged sword”, having both good and bad consequences for cancer [28, 29], and multiple factors including the category and stage of cancer, tumor microenvironment, and genetic context determine its role [30, 31].

### Tumor-suppressive role of autophagy

Autophagy, as an effective mechanism of senescence, is essentially important in protein remodeling to make an effectual transition of a proliferative tumor into a senescent form, and autophagic inhibition delays the senescence phenotype. Hence, autophagy might be critical in tumor suppression [32]. It prevents tumor induction, propagation, infiltration, and metastasis in the primary phase of tumorigenesis and functions as a tumor-suppressive process. It has well-known roles in cellular senescence, permanent arrest of cell division, and tumorigenic stress [33, 34, 35]. Intact autophagy has also shown its function as a tumor-suppressive mechanism either by downregulation of its genes or by a spontaneous increase in cancer malignancies because of the inefficiency of ATGs.

The tumor-suppressive role of autophagy is associated with deletions or mutations in a certain number of autophagy regulatory genes [36]. *BECN1*, a key gene in autophagy, encoding Beclin-1, is related to human cancer. Monoallelic deletion of *BECN1* has been observed as a tumor suppressor in ovarian and prostate cancers [37, 38]. Moreover, genes for multiple ATGs are linked to tumorigenesis. Frameshift mutations in *ATG2B, ATG5, ATG9B,* and *ATG12* downregulate autophagy in gastric and colorectal cancers [10]. The homozygote deletion and somatic point mutations of *ATG5* have also been identified as a tumor inducer in gastric and colorectal cancers and hepatocellular carcinoma [39, 40]. Another gene, *Bif-1*, involved in autophagosome formation, has been observed to downregulate in prostate and gastric cancers in mice [41]. Thus, intact autophagy plays a role in suppressing cancer by restricting genomic damage, preventing mutation, and blocking tumor initiation either through downregulation or gene deficiency.

Autophagy is also a key regulator of inflammation, a conventional feature of tumor progression in its early phase [42], and autophagy-deficient tumors exhibit a high level of necrosis and inflammation [43]. In defective autophagy, the failure to eliminate damaged polypeptides and cellular organelles prompts the dysfunction or death of the cell and subsequently encourages inflammation that leads to tumorigenesis by modifying the tumor microenvironment, raising oxidative stress, and inducing mutations that cause cancer [44]. ATG proteins along with Beclin-1 and LC3B are frequently studied for their roles in inflammation and cancer [45].

### Tumor inducer role of autophagy

In advanced stages of tumorigenesis, autophagy enhances the endurance and resistance of cancerous cells during stress and chemotherapy, mediates tumor growth and metabolism, and subsequently promotes tumorigenesis [46]. Cancerous cells are highly dependent on autophagy as compared with normal cells due to some intrinsic shortcomings in the microenvironment as well as excessive metabolic and biosynthetic requirements due to dysregulated proliferation. Cancerous cells are confronted by several stress factors, including hypoxia, nutrient deficiency, growth factor scarcity, pH fluctuations, and dysfunctional or inappropriate extracellular signals. To counter these unfavorable harsh conditions, cancerous cells mobilize autophagy, augment the resistance and survival of the cancerous cells against stress, and limit inflammation. So, tumors take over the survival function of autophagy to protect cellular damage and induce tumorigenesis under metabolic stress. Moreover, pathophysiological stimuli induce apoptosis, a pivotal cell death mechanism, to sustain cellular homeostasis and consecutive genetic mutations in cancerous cells. Under these circumstances, which are essentially important for cell survival, compromised or resistant apoptosis will lead to tumorigenesis [47].

Autophagy shows a multidimensional stage-specific role in cancer metastasis [48]. In the early stage of metastasis, it may suppress metastasis by prohibiting tumor necrosis and inflammatory cell infiltration. However, in later stages, it may support metastasis by endorsing diffusion of malignant cells in circulation, augmenting the entrance of metastatic cell debris in the targeted organs, and helping tumor cells to go into a dormancy state to protect them from the new environment [49, 50].

Autophagy is also boosted by p53 and Ras proteins, which are often mutated in human cancers. p53, a transcription factor, mediates the expression of a wide spectrum of genes. During starvation, a potent stimulus for autophagy, p53 suppresses the activation of LC3 via post-transcriptional modifications [51] and helps to promote cell survival by repressing autophagy. By contrast, an elevated level of p53 during stress conditions prompts the upregulation of certain genes that induce autophagy [52]. Therefore, whether p53 equips a pro- or antiautophagic signal in any described situation is context dependent. Ras, a small family of GTPases, has a regulatory role in cellular growth and survival [53]. Autophagy, with the assistance of H Ras and K Ras oncoproteins, plays a prosurvival role in tumor cells to help them cope with stress. Paradoxically, Ras-driven autophagy shows a prodeath role in cancer cells by upregulating the gene for Beclin-1. So, dependent upon the situation, Ras, with its downstream effectors, either promotes or inhibits autophagy and subsequent tumorigenesis [54, 55, 56, 57].

p62, a ubiquitin-binding scaffold protein, has been identified as a marker for autophagy inhibition or imperfection of autophagic degradation [58], and defective autophagy is machinery for p62 upregulation that is frequently monitored for in human tumors. Accumulation of p62 is directly correlated with cancer progression, and autophagy prevents the accumulation of p62 and suppresses tumorigenesis [59, 60].

### Autophagy modulation in cancer therapy

**Figure 2:**
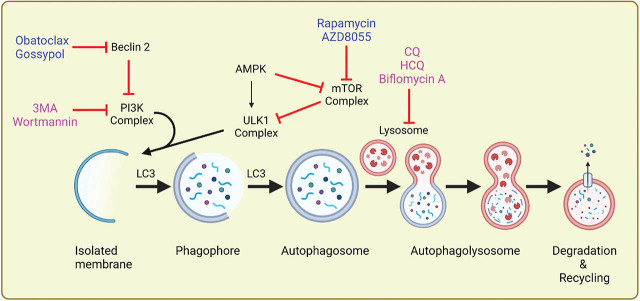
Schematic of macroautophagy flux. Autophagy starts with the development of a phagophore followed by the nucleation and elongation controlled by a class III PI3K complex and subsequently enclosure of cytoplasmic contents or organelles to form an autophagosome. The autophagosome then merges with a lysosome to develop into an autophagolysosome, the ultimate site for degradation and recycling. mTOR negatively controls autophagic flux, and it is inhibited by rapamycin and AZD8055 to induce autophagy. Beclin-2 also negatively controls autophagy by inhibiting the PI3K complex, and obatoclax and gossypol inhibit Beclin-2. 3-Methyladenine and wortmannin are early-stage inhibitors that hinder autophagy by negative control of the class III PI3K, and chloroquine, hydroxychloroquine, and biflomycin A are late-stage inhibitors that block the autophagic flux by interfering with the lysosome directly. Created with BioRender.com. Academic License DS23ZNI6O2. mTOR, mammalian targets of rapamycin; PI3K, phosphatidylinositol 3-kinase; CQ, chloroquine; HCQ, hydroxychloroquine.

Autophagy is triggered by the actions of various signaling pathways of the cell, autophagy-related proteins, and their complexes engaged in autophagic flux (**Figure 2**) [61, 62].

Ascertaining whether autophagy is cytoprotective or cytotoxic assists in defining approaches for its modulation and antineoplastic regimens, including chemotherapy and radiotherapy. Autophagy modulators may improve the effectiveness of cancer therapy when combined with conventional treatments [63, 64, 65, 66, 67].

The modulation of autophagy is an encouraging approach to cancer therapy [67]. Inhibition of cytoprotective autophagy may improve response in cancer treatment [68, 69]. Various drugs have been recognized to target autophagy, from induction to degradation and recycling, either by inhibiting the regulatory complexes and proteins necessary to initiate autophagy or by targeting the lysosome directly (**Table 1**). 3-Methyladenine and wortmannin are early-stage autophagy inhibitors that suppress autophagy through the inhibition of the PI3K complex and prevent the formation of autophagosomes [70]. 3-Methyladenine can also upregulate p62 protein expression at the mRNA level that plays a major role in autophagy, apoptosis, and cancer development [71]. However, the inhibitory role of 3-methyladenine is controversial as it may promote autophagic flux in nutrient-rich conditions, thus exerting a dual role in autophagy [72]. Moreover, 3-methyladenine can target other kinases and interfere with other processes at the cell level, such as glycogen metabolism, change in pH of the lysosome, and endocytosis [73].

**Table 1:** Autophagy regulators used in cancer therapy

**Autophagy regulator**	**Type**	**Mode of action**		**References**
3-Methyladenine	Inhibitor	P13K inhibitors	Upregulation of p62 protein expression	[60, 70, 71, 72]
Wortmannin			Inhibit phagosome formation	[70, 73, 74]
LY294002			Promote apoptosis	[71, 75]
Chloroquine		Lysosome inhibitors	Prevent acidification	[76]
Hydroxychloroquine			Inhibit autophagosome accumulation Inhibit the formation of autolysosome	[77, 78, 79, 80]
Bafilomycin A			Inhibit autophagic degradation	[81]
Tioconazol		ATG inhibitors	Inhibit phagophore elongation	[82]
FMK-9a			Inhibit autophagosome fusion	[83]
Rapamycin	Inducer	mTOR inhibitors	Prevent inactivation of ULK1 phosphorylation	[84]
AZD8055			Activate or induce autophagy Induction of apoptosis Cell cycle arrest	[85, 86]
Vitamin D		Natural products or mTOR inhibitors	Increase intestinal calcium absorption Induce expression of proapoptotic proteins Induces an autophagic transcriptional signature Induce autophagosome formation	[87, 88]
Resveratrol			Induce apoptotic cell death Suppress growth of cancer cell	[89]
Curcumin			Induce apoptosis Inhibit phosphorylation of AKT	[90]
Obatoclax		BH3 Mimetics or Beclin-2 inhibitors	Bcl-2 inhibition	[91, 92]
Gossypol			ROS production Autophagic-mediated necroptosis	[93]

AKT, protein kinase B; mTOR, mammalian targets of rapamycin; ATG, autophagy-related genes; ROS, reactive oxygen species; ULK1, Unc-51 like autophagy activating kinase 1.

Inhibition of autophagy can also be accomplished by blocking ATG proteins, which show a promising role from the elongation of phagophores to the formation of autophagolysosomes [82]. Inhibitors of ATG4 and ATG7 have been used to modulate autophagy in cancer therapy [82, 83]. Like these regulatory complex inhibitors, the lysosomal inhibitors, chloroquine and hydroxychloroquine, have been commonly used in combination with chemotherapies to treat multiple myeloma, brain, lung, kidney, or prostate cancer. They modulate autophagy via acidification of lysosomes and inhibit autophagosome accumulation [79]. Another potential inhibitor of autophagy, bafilomycin A, has been used to increase apoptotic cell death after radiotherapy. Bafilomycin A restricts autophagy by altering the pH of the lysosome and preventing autophagosome–lysosome fusion. Thus, the generation of anticancer agents that overwhelmed resistance to anticancer therapy has been prompted [94]. Moreover, these reagents upregulate the therapeutic response by suppressing autophagy-mediated resistance to cancer therapy [95]. Although inhibiting autophagy with these compounds has been explored in clinical trials, still there is a room for upgradation because chloroquine and hydroxychloroquine have also been exposed to have noteworthy unpredictability in inhibiting autophagy in patients. Consistent with the pharmacological use of these autophagy inhibitors, silencing or activation of autophagy genes can also increase tumor cell sensitivity to autophagy [67, 96].

By contrast, induction of cytotoxic autophagy might be helpful to improve the effectiveness of anticancer therapies by inducing cell death itself or by activating apoptosis [97, 98]. Several drugs and natural extracts have been reported to trigger autophagy-regulated cell death in various cancerous cells [99] by suppressing the complexes having an inhibitory effect in autophagy (Table 1). mTOR regulates autophagy by inhibiting its initiation. Rapamycin along with its semisynthetic analogs (rapalogs) are allosteric inhibitors of mTORC1 that target the activation of autophagy by preventing the inactivation of ULK1 phosphorylation. However, the efficiency of rapamycin as a tumor inhibitor is limited as it cannot inhibit mTORC2 or certain other compensatory pathways that enhance cell endurance [100]. AZD8055 is another mTOR inhibitor that induces autophagy by inhibiting both mTOR complexes and preventing tumor growth in hepatocellular carcinoma cell lines [85, 101].

Some natural products have also earned a noteworthy reputation as safe and effective compounds that can be used to target autophagy [102]. Vitamin D is a major regulator that induces autophagy through calcium metabolism [88]. A high level of circulating vitamin D leads to increased intestinal calcium absorption and induces autophagy by activating calcium-dependent kinases and phosphatases. It can also down-regulate mTOR protein expression and induce autophagy as an mTORC1 inhibitor. Moreover, vitamin D mediates apoptosis by destabilizing telomerase reverse transcriptase (TERT) mRNA, resulting in the downregulation of telomerase activity in epithelial ovarian cancer cells [103]. Resveratrol and curcumin are some natural products that have been reported to induce autophagy by inhibiting phosphorylation of protein kinase B (AKT) and induce apoptotic cell death and suppress the growth of cancer cells [104]. Like these mTOR inhibitors and natural products, BH3 mimetics imitate the interaction of BH3 proteins and induce autophagy by inhibiting Bcl-2. Gossypol and obatoclax are BH3 mimetics that have a high affinity for Bcl-2 and are used to induce autophagy in several kinds of cancers [105]. Additionally, nanoparticles such as gold and zinc oxide are used for targeted delivery of autophagy regulators and reduce cancer progression by enhancing apoptosis and autophagy [106]. Moreover, a phototherapy-based approach, photothermal therapy (PTT), is a promising alternative treatment to suppress tumor growth by activating autophagy or suppressing the cell signaling pathways that effect cell cycle and subsequently induce cell death [107].

Overall, the clinical data on autophagy modulation in cancer therapy show a promising approach to overcome resistance to chemotherapy and radiotherapy and enhance the outcomes of these cancer treatments, but this approach requires a comprehensive understanding of the response and the mode of action of these modulators.

### Future perspectives

Autophagy is a key process to counteract the stress response to chemotherapeutic drugs and radiation by cancer cells. It acts as a double-edged sword, where on the one hand autophagy protects tumor cells from cancer therapy, while on the other, it might also kill cancer cells. Therefore, it is of prime importance to elucidate the tumorigenic essentials for autophagy to rationalize the use of autophagy inhibition or activation with the goal of enhancing the therapeutic response. Consistent with this, most of the cancer models developed to date have focused on understanding the role of autophagy in cancerous cells and not directly compared its deficit in normal tissues. Because it is an essential process for some normal tissues, an essential question is whether inactivation of autophagy will necessarily be sufficiently selective to disrupt the growth of cancerous cells, while sparing normal tissues from harmful effects. Targeting critical modules of the autophagic machinery may be a prerequisite to determine whether or not this method is therapeutically helpful.
